# Efficacy of nano encapsulated herbal extracts in the treatment of induced wounds in animal models: a systematic review protocol

**DOI:** 10.1186/s13643-023-02370-7

**Published:** 2023-11-16

**Authors:** Catherine Namuga, Moses Ocan, Alison A. Kinengyere, Ssenono Richard, Eve Namisango, Haruna Muwonge, John Baptist Kirabira, Mugisha Lawrence, Ekwaro A. Obuku

**Affiliations:** 1 Department of Polymer, Textile and Industrial Engineering, Busitema University, Tororo, P. O. Box 256, Uganda; 2 Department of Mechanical Engineering, Makerere University, College of Engineering, Design, Art and Technology, Kampala, P.O. Box 7072, Uganda; 3 Department of Pharmacology, Makerere University College of Health Sciences, School of Biomedical Sciences, P.O. Box 7072, Kampala, Uganda; 4 Department of Medicine, Africa Centre for Knowledge Translation and Systematic Reviews, College of Health Sciences, Makerere University, P.O. Box 7072, Kampala, Uganda; 5 Albert Cook Library, Makerere University, P.O. Box 7072, Kampala, Uganda; 6 Infectious Diseases Institute, Makerere University, P. O. Box 22418, Kampala, Uganda; 7 Clinical Epidemiology Unit, Department of Medicine, School of Medicine, Makerere University College of Health Sciences, Kampala, Uganda; 8 Department of Physiology, Makerere University, College of Health Sciences, P. O. Box 7072, Kampala, Uganda; 9 Department of Physiology, Habib Medical School, Islamic University in Uganda (IUIU), P.O Box 7689, Kampala, Uganda; 10 College of Veterinary Medicine, Animal Resources and Biosecurity, Makerere University, P. O. Box 7072, Kampala, Uganda

**Keywords:** Wounds, Wound healing, Herbal extracts, Nanoencapsulation, Nanocarriers

## Abstract

**Background:**

Wounds inflict pain and affect human health causing high expenditure on treatment and management. Herbal crude extracts are used in traditional medicine as a treatment for wounds and other illnesses. However, the progress in the use of plants has been deterred due to their poor solubility and poor bioavailability requiring administration at high doses. It has been established that nanoencapsulation of herbal products in nanocarriers (size 1 nm to 100 nm) such as nanofibers, nanoparticles, nanospheres, and nanoliposomes greatly improves their efficacy. Due to their small and large surface area, nanocarriers are more biologically active, improve bioavailability, protect the drug from deterioration, and release it to the targeted site in a sustainable manner.

**Aim:**

The review aims to collate and appraise evidence on the efficacy of nano encapsulated herbal extracts in the treatment of induced wounds in animal models.

**Methods:**

The review will be protocol-driven and conducted according to the Preferred Reporting Items for Systematic Reviews and Meta-analysis for Protocols (PRISMA-P) and protocol guidelines for systematic review and meta-analysis for animal intervention studies. The final review will be conducted and reported with reference to PRISMA 2020 statement. Studies will be searched in Pub Med, ProQuest, Web of Science, Medline Ovid, EMBASE, and Google Scholar. The PRISMA flow criteria will be followed in screening the articles for inclusion. Data extraction form will be designed in Excel spreadsheet 2013 and data extracted based on the primary and secondary outcomes. Risk of bias assessment will be done using SYRCLE’s risk of bias tool for animal studies. Data analysis will be done using narrative and quantitative synthesis.

**Expected results:**

We hope to make meaningful comparisons between the effectiveness of the herb-loaded nanomaterials and other interventions (controls) in the selected studies, based on the primary and secondary outcome measures. We expect that these findings to inform clinical practice on whether preclinical studies show enough quality evidence on the efficacy and safety of herbal-loaded nanomaterials that can be translated into clinical trials and further research.

**Systemic review registration:**

PROSPERO 330330. The protocol was submitted on the 11th of May 2022.

**Supplementary Information:**

The online version contains supplementary material available at 10.1186/s13643-023-02370-7.

## Background

Wounds inflict pain and seriously affect the patient’s state of well-being with medical care and treatment coming at a huge expense. In the USA, the healthcare cost of wounds accounts for about 28.1–31.7 billion USD per year, and the prevalence of chronic wounds is projected to increase due to diabetes, obesity, and other diseases [1]. Infection is the leading cause of delayed healing, and this largely increases the cost of wound care. In Europe, out of 10,000 operations performed in a hospital, 3 to 4% result in infected wounds, requiring a treatment cost of approximately two million euros per year [2]. Of hospital admissions in developed countries, 50% are due to surgical wound infection, while in developing countries, the problem is underrated [3]. In Uganda, the cost of wound care is not well ascertained since not all patients report to the hospital however, it was reported that 10% of the surgeries performed in Uganda become infected leading to hospitalization and mortality [4].

A wound is a disruption of cellular and anatomic continuity of tissue, caused by physical, microbial, thermal, chemical, or immunological tissue trauma [5]. Wounds may be classified as acute or chronic, open or closed, [2, 6, 7] superficial, full-thickness, or partial-thickness wounds [8]. Wound healing involves complex processes that occur simultaneously in four stages: hemostasis, inflammation, proliferation, and remodeling [9]. Wounds should heal naturally; however, in some cases, healing is prolonged due to factors such as infection, poor surgical techniques, tissue ischemia, poor nutrition, vitamin deficiency, aging, as well as underlying diseases [6, 8]. Patients with diabetes and other underlying diseases are at risk of non-healing wounds, and sadly, most of the currently used interventions in wound management have not been effective enough [10]. Therefore, the quest to find novel and better interventions continues.

Wound treatment is often preceded by debridement, followed by exudate control, activating wound healing, and wound protection using dressings [11]. Wound infections are treated using antibiotics which may be administered orally or directly on the wound or incorporated into the dressing and delivered to the wound site [8]. However, some common antibiotics used in the treatment of wounds have less microbial coverage, are toxic, and have poor permeability leading to antimicrobial resistance and failure to improve the proliferative phase [12]. Aside from synthetic antibiotics, herbal medicines have become of great therapeutic value in the treatment of infections and other illnesses [13]. Of the developing and underdeveloped countries, 80% still utilize plant herbs in primary healthcare [5]. In fact, of all modern medicine on the worldwide market today, more than 60% has been indirectly or directly developed from herbs and other natural materials [14]. Plants naturally contain phytocompounds that possess antimicrobial, antioxidant, and anti-inflammatory characteristics which are essential for wound healing [15]. The phytochemicals work synergistically using different mechanisms to facilitate the healing process. However, most of the phytocompounds are hydrophobic and have poor solubility. Poor dissolution decreases their bioavailability which requires the drug to be administered in high doses and treatment is prolonged [16–18]. Moreover, they are unable to penetrate through lipid membranes of cells having larger molecular sizes. As a result, their efficacy and bioavailability are lost [19].

Encapsulation of plant extracts into nanomaterials is one magical way to solve this problem. Nanoencapsulation deals with synthesizing drug-loaded structures having diameters between 1 and 1000 nm. Nanocarriers can encapsulate the herbal drug into their structures so that they are released sustainably to the targeted biological site [12], and interact with the targeted site for a long time, with minimal side effects and dosage [20]. When plant extracts are loaded into these nano delivery systems, their toxicity is minimized, solubility and bioavailability increase, efficacy is greatly improved, the extract is protected from degradation, and better still, they are delivered to the targeted site in a sustainable manner. For herbal drug delivery, nanocarriers such as liposomes, polymeric nanoparticles, solid lipid nanoparticles, proliposomes, and nanoemulsion are the most suitable and effective for this purpose [21]. These may be incorporated into other structures such as hydrogels and nanofibers. With nanofibers, the extract may be directly loaded into the nanofiber or encapsulated in the nanoparticle and later loaded into the nanofiber during the electrospinning process [15].

Wound healing models are important in studying the pathogenesis of healing and scar formation as well as testing new therapeutics. Models utilized in research include animal models, in silico, in vitro, and human models (ex vivo and in vivo) [22]. In vivo animal models are the most efficient and clinically relevant when studying wound healing because the study of pathophysiology of wound healing is enabled unlike in vitro and in silico. Although animal skin does not resemble human skin, they have been developed and utilized in order to study the complexity of the wound healing process before clinical trials [23]. Studies using animal models should focus on clinical relevance, proper interpretation of quantitave data, results be reproducible, and lead to successful transition to clinical trials and later clinical practice. Animals used in most studies are rodents, rabbits, and pigs. Rodents are the most common due to their small size, ease of maintenance, and cost effectiveness [24]. The most commonly used animal wound models in pharmacological studies include:

i. Excision wound model: The wound is created on the dorsal part of the animal. The wound contraction area, wound index, collagen formation, hydroxyproline content protein estimation, and histopathology can all be assessed using this model [23].
ii. Incision wound model: An incision of about 2 mm depth is made on the back of the animal and later sutured. The most assessed parameter is the wound tensile strength which is measured after the sutures are removed [23].
iii. Burn wound model: Ten-millimeter diameter burn wound is created using a hot cylindrical metallic rod. The most sought out parameters are period of epithelization and wound contraction rate [23].
iv. Dead space wound model: a transverse incision is made on the dorsal paravertebral skin region of the animal. Parameters evaluated are estimation of protein and DNA, tensile strength, hydroxyproline, and hexuronic content as well as the amount of hexosamine [23].

### Rationale for the review

It has been established in several studies that nano herbal drug delivery has proved to be of great therapeutic value in accelerating the healing of wounds. Some narrative reviews have been done in regard to the effectiveness of nanophytomedicines for wound healing and report promising results. Qadir et al. [25] conducted a narrative review on effectiveness of phytomedicine-based nanopharmaceuticals on burn wound healing. A review by Hajialyani et al. [26] focused on a broad scope encompassing all-natural plant-based nanomedicines (whether nanoencapsulated or not) for wound healing including green synthesized nanoparticles. Both reviews found that various plant products used in nano form improve wound healing. However, they do not clearly state the methods in which the quality of evidence was assessed, and the scope was wide. To the best of our knowledge, there is no systematic review that has systematically appraised the quality of evidence across literature, with a focus on the effectiveness of nanoencapsulation of herbal extracts on wound healing invivo.

This systematic review’s focus is to critically evaluate the quality of evidence available on efficacy and safety of nano encapsulated herbal extracts as the main intervention for wound healing in animal models, to enable the transition to clinical research. The review focusses on studies that have evaluated nano delivery of herbal extracts for wound healing using animal wound models because other wound assessment methods such as in silico and in vitro do not involve other matrix tissue contents involved in wound healing [27]. Furthermore, with the use of skin explants (ex vivo), the desquamation of cells cannot be observed, and more so, innervation is limited, yet, it is crucial in understanding skin repair and scar formation [22]. However, in animal wound models, the study of pathophysiology of wound healing is enabled unlike in vitro. Although animal skin does not resemble human skin, they have been utilized in order to study the complexity of the wound healing process before clinical trials [23]. They can provide reliable and reproducible information on the behavior and response of wounds to experimental therapy. They also serve as a valuable research tool in the search for faster, stronger, and more anatomically correct wound healing with the ultimate goal of exact skin replacement [28].

### Review objectives

The review seeks to generate evidence-based studies of preclinical trials that support the effectiveness of nanoencapsulation of herbal extracts in accelerating wound healing in animal wound models.

The review also seeks to determine whether there are nanocarriers that lead to better wound healing outcomes than others.

The review will ascertain whether there is sufficient quality of evidence on the efficacy of herb-loaded nanocarriers to enable the transition from preclinical trials to clinical trials.

## Methods

### Protocol development

This review will be performed following guidelines from the Preferred Reporting Items for Systematic Reviews and Meta-analysis for Protocols (PRISMA-P) (Additional file 1) [29] and the protocol guidelines for Systematic review and meta-analysis for animal intervention studies [30]. The final review will be conducted and reported with reference to PRISMA 2020 statement [29] and published in a peer-reviewed journal. This protocol has been submitted for registration in PROPSERO (https://www.crd.york.ac.uk/prospero/) (Application number 330330).

### Review question

Is there a difference between the percent wound closure of induced wounds in animal wound models treated with nano encapsulated herbal extracts and that of other alternatives used? The PICOS model for the review question is shown in Table 1.

**Table 1:** PICOS model for the review question

PICOS element	Description of the element
Population	Rat and mice, rabbits, and pigs/porcine with induced wounds (excision wounds, incision wounds, cutaneous wounds, burn wounds, diabetic wounds, splint wounds, or any other wound model)
Intervention	Any nanocarrier that has been incorporated/encapsulated with herbal extracts (crude extracts, isolated phytochemicals, plant oils) to enhance wound healing. For example, polymeric nanoparticles, liposomes, solid lipid nanoparticles, proliposomes and nanoemulsion, nanocomposites, nanogels/hydrogels, and nanofibers
Comparator	Herbal extract that has not been encapsulated into the nanocarrier, nanocarrier without the extract, standard antibiotic, or dressing, placebo
Outcome	Primary outcome: Rate of wound contraction (%wound closure) Secondary outcome: Rate of re-epithelization, increase in tensile strength, histopathological results (granulation tissue formation, cell proliferation, neovascularization, collagen synthesis), hydroxyproline, hexosamine, and hexuronic content, whether healing left a scar or not, immunohistochemical analysis
Study design	Experimental laboratory design using animal wound models

### Article search

Articles on the efficacy of herb-loaded nanomaterials on in vivo wound healing published from 2000 (there is no available data before this year) to date will be searched from the following electronic databases: Web of Science, MEDLINE Ovid, Pub Med, EMBASE, and Google Scholar.

The following grey literature sources will also be searched: The Agency for Healthcare Research and Quality (AHRQ), Bielefeld Academic Search Engine (BASE), National Institute for Health and Care Excellence (NICE), Networked Digital Library of Theses and Dissertations (NDLTD), Open Grey, ProQuest Dissertations & Theses, National Library of Medicine (NLM), and World Health Organization Institutional Repository for Information Sharing (WHO IRIS).

### Search strategy

The search will be conducted by a qualified librarian, Alison A. Kinengyere, as an information retrieval specialist. An electronic search will be conducted with the following search terms and their Medical subject heading (MesH) in abstract, keyword, and text.

- Animal (rats or mice or rabbits or pigs) to represent the population.
- Plant (herb or crude extract or plant extract or herbal medicine or phytochemical or leaf extract or essential oil) as the main intervention.
- Nano (nanoparticles or nano-composites or nano-capsules or nano-spheres or nano-medicine or nano-liposomes or nanofibers or nanomaterials or nano-gels or dendrimers or cyclodextrins). To capture aspects of the intervention.
- Wound healing, (wound or wound model or excisional wound or incision wound or murine wound or dead space or burn wound or infected wound or diabetic wound or splinted wound or cutaneous wound) to capture aspects of the outcome.

The search terms will be combined using Boolean logic “OR” for related terms and “AND” for terms from different elements of PICOS. Truncation and wildcards will be added to terms where applicable.

A stepwise serial search pilot example from PubMed is provided (See Additional file 2). Once papers are included, the reference lists of those retrieved papers will be checked for additional eligible studies. In addition, authors will be contacted to get the missing information. The retrieved articles will be imported using endnote software and duplicates removed.

### Selection criteria

Articles will be screened for inclusion and exclusion according to the following criteria:

#### Study design

- *Inclusion*: experimental laboratory design setting using animal wound models. Studies that compare with control group.
- *Exclusion*: studies without controls.

#### Population

- *Inclusion*: Animal (rats, mice, rabbits and pigs/porcine) with laboratory induced wounds will be included.
- *Exclusion*: wound healing studies in vitro, ex vivo, in silico, or in humans.

#### Intervention

- *Inclusion*: Studies that evaluate wound healing where herbal crude extract or phytochemical or plant oil is encapsulated inside the nanomaterial.
- *Exclusion*: Studies involving plant synthesized metallic nanomaterials. Studies on wound healing using any herbal crude extracts or phytochemicals or oils that have not been encapsulated into a nanocarrier will not be considered.

#### Outcome measure

- *Inclusion*: Studies where wound healing has been evaluated will be considered.
- *Exclusion:* outcomes other than those related to wound healing will be excluded.

#### Others

- *Inclusion:* only original peer-reviewed papers in the English language published from the year 2000 to date.
- *Exclusion*: case reports, conference papers, reviews, editorials, unpublished work, and letters to the editor.

### Screening and selection of articles

The literature will be retrieved from the listed databases using the mentioned search strategies and the findings reported using the PRISMA 2020 statement [29]. Endnote reference management software will be used to manage citations, bibliographies, export and import citations, as well as de-duplicate retrieved studies. We will contact the authors of retrieved studies for missing data or additional data, where required.

Groups of included and excluded studies will be created in endnote as follows:

- Group 1 will be named *included studies*, where all studies that meet the inclusion criteria will be put.
- Group 2 will be named *excluded studies*, where all studies that do not meet the inclusion criteria will be put.
- Group 3 will be named *unresolved*, where studies awaiting a tiebreaker to resolve will be put.
- The three folders will be used during Title/Abstract screening. After Title/Abstract screening, a fourth folder will be created and named *included final*, where studies that meet the inclusion criteria after full-text screening will be put.

Two review team members will independently screen titles and abstracts of retrieved studies to identify studies that potentially meet the inclusion criteria.

Before obtaining the full text of retrieved studies or literature, the results of this screening process will be compared and discussed to reach a consensus concerning the studies to be obtained in full-text format.

Two review team members will independently review the full text of studies whose titles and abstracts have been screened eligible using the inclusion criteria. Any disagreements that may arise among the review members will be resolved by consensus or with reference to a third team member as appropriate.

### Data extraction

We will develop standardized data extraction spreadsheet forms in Excel. Three team members will independently extract data from included studies onto the form. Data extraction forms will be used in extracting data from the selected studies with the help of the ARRIVE (Animal Research: Reporting In Vivo Experiments) guidelines [31]. The abstracted data will be kept in Excel 2013 file. The form will be piloted on five studies to check for expected review outcomes, and basing on the findings, adjustments will be made. The level of agreement between reviewers will be determined by the Kappa statistic (> 0.75 for excellent, 0.40–0.75 moderate, and < 0.40 for poor) [32]. Any further disagreements will be referred to a tiebreaker. Extracted data will mainly be based on the key PICO components addressing our research question. Information that will be extracted from studies includes:

- Study ID: country, author, year of publication, publication status, journal of publication.
- Population: animal (type of animal, strain, species, gender, weight, housing, and feeding conditions).
- Condition of the wound: wound model, wound type, location, wound size, severity, infected or non-infected.
- Intervention: formulation, concentration, dose, administration route, administration frequency, duration, type and formulation of the nanocarrier.
- Comparator: number of controls, type of controls.
- Outcome measure: wound contraction rate (%wound closure), tensile strength, histopathology results, the number of days to complete healing, whether healing left a scar or not, immunohistochemical analysis.
- Study design: sample size calculation, sample size, number of experimental units, number of animals per group, sampling methods.
- Randomization methods: whether blinding was done and at what stages it was done.

We will also find out whether there are animals that were excluded from the analysis (dropouts) and reasons for their exclusion.

In incidences where the study used multiple interventions, only the data relating to our research question will be extracted. Data will be extracted from graphs, text, and tables. Graphical data will be extracted using digital screen ruler [33]. Where necessary, we shall contact the authors for missing information.

### Outcome measure

Our primary outcome will be the wound healing rate expressed as the mean percentage proportion of a completely healed wound (wound closure) defined as %Wound closure = $\frac{A0-An}{A0}$×100 (where; $A0$ is the area of the wound at day 0, and $An$ is the area of the wound on the *n*th day after wound induction).

Secondary outcome measures will include the following: the rate of re-epithelization, the time to complete wound healing, wound tensile strength, histopathology (granulation tissue formation, re-epithelization, neovascularization, fibroblast proliferation, collagen synthesis, presence of hair follicles, inflammatory response), hydroxyproline, hexosamine and hexuronic content, and immunohistochemical analysis.

### Quality assessment

We anticipate that all included studies will use experimental laboratory design using animal models. Therefore, we will use the Systematic Review Centre for Laboratory Animal Experimentation (SYRCLE) risk of bias tool for animal studies [34] to assess the data quality and risk of bias. The tool was derived from the Cochrane Collaboration Risk of Bias Tool. It is adapted to assess methodological quality and features of bias such as selection bias, performance bias, attrition bias, and reporting bias [34]. Two review members will assess the risk of bias. Any disagreements will be resolved through discussion with the involvement of a third party. Two team members will individually assess the quality of evidence across all the included studies for the outcome of interest. They will use the Grading of Recommendations, Assessment, Development, and Evaluations (GRADE) approach [35] for summary assessment of the certainty of evidence. Any discrepancies will be resolved by discussion and consensus or referral to a third member if necessary.

### Data analysis

A quantitative and narrative synthesis of the studies included will be presented using a descriptive data table. We will report the experimental outcomes of the studies. These will include study setting, study design, population, intervention, outcome measures, complications or adverse effects, and any other suitable findings of each study. In this narrative evaluation, we shall comment on whether the efficacy of herb-loaded nanomaterials on wound healing appears to vary according to the intervention subgroups. If the findings are suitable for meta-analysis, we shall first take a heterogeneity analysis related to study design, population, interventions, comparators, and outcomes.

Dichotomous data will be analyzed using risk ratios, while continuous data will be analyzed using mean differences or standard mean differences. Statistical heterogeneity will be assessed using chi square and quantified using the *I*^2^ statistic. The threshold for *I*^2^ value will be interpreted as follows: 0–25% for very low heterogeneity, 25–50% for low heterogeneity, 50–75% moderate heterogeneity, and above 75% for high heterogeneity [36]. We shall use random-effects model because of the nature and differences in animal studies [33]. Subgroup analyses will be performed to further interrogate primary and/or secondary outcomes based on the type of nanocarrier (nanoparticle, nanoliposome, nanocomposite, nanofiber, nanoemulsion, hydrogel). To also ascertain whether either of the main intervention types (i.e., crude extract, isolated phytochemical, essential oil) is superior, we will present the data according to the herbal intervention used if sufficient data are available. If the findings of the meta-analysis are robust, we shall perform a sensitivity analysis [33]. The analysis will done in STATA 15 and CAMARADES data-manager. Publication bias will be assessed using funnel plots as well as trim and fill if necessary.

## Discussion

The use of herbal medicine has taken place way back in history. With the current concern in antibiotic resistance, more attention is being paid to herbal therapeutics. However, their use has posed various challenges, especially their poor bioavailability and solubility that limit their efficacy. With the emergence of nanotechnology, there is hope that the challenges faced with herbal medicine will be solved resulting in the formation of novel and effective herb-loaded therapeutics. Thus, a systematic appraisal of available evidence on the wound healing effect of herb-loaded nanomaterials is felicitous.

Preclinical trials of new interventions using animal models are always necessary and provide evidence on whether a new intervention may proceed for clinical trials in humans without posing adverse effects. Therefore, in this review, we hope to establish evidence as to whether incorporating herbal medicine into nanocarriers improves their therapeutic effect on wounds. Based on the data retrieved, we hope to find out which nanocarrier is more effective for herbal delivery in wound healing and whether this may vary according to the type of wound or the type of herb used. We also expect to establish whether loading the herb into the nanocarrier in the form of an isolated compound (phytochemical) or essential oil may be better than using a crude herbal extract. Additionally, we hope to make meaningful comparisons between the effectiveness of the herb-loaded nanomaterials and other interventions used as controls in the selected studies, based on the primary and secondary outcome measures. We hope that all these findings will be able to inform clinical practice and other researchers on which nanocarrier is most suitable for herbal delivery and can proceed for clinical trials and further research. We shall discuss all the relevant nanocarriers involved in nanoencapsulation of herbal extracts, as well as the areas of uncertainty within the current literature in a way that we can guide further research. We will assess the strength of our conclusions using the validated methodology for appraising the quality of evidence for each summary of outcomes.

### Supplementary Information

**Additional file 1.** PRISMA-P 2015 Checklist.

**Additional file 2.** Pilot search strategy.

## Data Availability

Available data: Search strategy for PubMed (Attached file 2), PRISMA-P checklist (Attached file 1).

## Abbreviations

PRISMA: Preferred Reporting Items for Systematic Reviews and Meta-analysis
SYRCLE: Systematic Review Centre for Laboratory animal Experimentation
ARRIVE: Animal Research: Reporting In Vivo Experiments
GRADE: Grading of Recommendations, Assessment, Development, and Evaluations
CAMARADE: Collaborative Approach to Meta-Analysis and Review of Animal Experimental Studies

## References

[1] O'Callaghan S, Galvin P, O'Mahony C, Moore Z, Derwin R (2020). ‘Smart’wound dressings for advanced wound care: a review. J Wound Care.

[2] Andreu V, Mendoza G, Arruebo M, Irusta S (2015). Smart dressings based on nanostructured fibers containing natural origin antimicrobial, anti-inflammatory, and regenerative compounds. Materials.

[3] Lubega A, Joel B, Justina Lucy N (2017). Incidence and etiology of surgical site infections among emergency postoperative patients in mbarara regional referral hospital, South Western Uganda. Surg Res Pract.

[4] Seni J, Najjuka CF, Kateete DP, Makobore P, Joloba ML, Kajumbula H (2013). Antimicrobial resistance in hospitalized surgical patients: a silently emerging public health concern in Uganda. BMC Res Notes.

[5] Maver T, Maver U, Stana Kleinschek K, Smrke DM, Kreft S (2015). A review of herbal medicines in wound healing. Int J Dermatol.

[6] Ather S, Harding K, Tate S (2019). Wound management and dressings.

[7] Singh AV, Aditi SA, Gade WN, Vats T, Lenardi C, Milani P (2010). Nanomaterials: new generation therapeutics in wound healing and tissue repair. Curr Nanosci.

[8] Boateng JS, Matthews KH, Stevens HN, Eccleston GM (2008). Wound healing dressings and drug delivery systems: a review. J Pharm Sci.

[9] Voncina B, Fras LZ, Ristic T. Active textile dressings for wound healing. Advances in Smart Medical Textiles. Elsevier; 2016. p. 73–92.

[10] Rodrigues M, Kosaric N, Bonham CA, Gurtner GC (2019). Wound healing: a cellular perspective. Physiol Rev.

[11] Rajendran S, Anand SC. 6 - Advanced textiles for wound compression. In: Rajendran S, editor. Advanced Textiles for Wound Care. Woodhead Publishing; 2009. p. 153–78.

[12] Monika P, Chandraprabha M (2020). Phytonanotechnology for enhanced wound healing activity.

[13] Bonifácio BV, da Silva PB, dos Santos Ramos MA, Negri KMS, Bauab TM, Chorilli M (2014). Nanotechnology-based drug delivery systems and herbal medicines: a review. Int J Nanomed.

[14] Kiguba R, Ononge S, Karamagi C, Bird SM (2016). Herbal medicine use and linked suspected adverse drug reactions in a prospective cohort of Ugandan inpatients. BMC Complement Altern Med.

[15] Shah A, Amini-Nik S (2017). The role of phytochemicals in the inflammatory phase of wound healing. Int J Mol Sci.

[16] Namdari M, Eatemadi A, Soleimaninejad M, Hammed AT (2017). A brief review on the application of nanoparticle enclosed herbal medicine for the treatment of infective endocarditis. Biomed Pharmacother.

[17] Huang S, Chang WH (2009). Advantages of nanotechnology-based Chinese herb drugs on biological activities. Curr Drug Metab.

[18] Ansari S, Farha IM (2012). Influence of nanotechnology on herbal drugs: a review. J Adv Pharm Technol Res.

[19] Pattabhiramaiah M, Rajarathinam B, Shanthala M (2020). Nanoparticles and their application in folklore medicine as promising biotherapeutics.

[20] Mordorski B, Prow T (2016). Nanomaterials for wound healing. Curr Dermatol Rep.

[21] Das S, Sharangi AB (2020). Nanotechnology: a potential tool in exploring herbal benefits.

[22] Sami DG, Heiba HH, Abdellatif A (2019). Wound healing models: a systematic review of animal and non-animal models. Wound Med.

[23] Shrivastav A, Mishra AK, Ali SS, Ahmad A, Abuzinadah MF, Khan NA (2018). In vivo models for assesment of wound healing potential: a systematic review. Wound Med.

[24] Grada A, Mervis J, Falanga V. Research techniques made simple: animal models of wound healing. J Investig Dermatol. 2018;138(10):2095–105. e1. 10.1016/j.jid.2018.08.005 30244718

[25] Qadir A, Jahan S, Aqil M, Warsi MH, Alhakamy NA, Alfaleh MA (2021). Phytochemical-based nano-pharmacotherapeutics for management of burn wound healing. Gels.

[26] Hajialyani M, Tewari D, Sobarzo-Sánchez E, Nabavi SM, Farzaei MH, Abdollahi M (2018). Natural product-based nanomedicines for wound healing purposes: therapeutic targets and drug delivery systems. Int J Nanomed.

[27] Ud-Din S, Bayat A (2017). Non-animal models of wound healing in cutaneous repair: in silico, in vitro, ex vivo, and in vivo models of wounds and scars in human skin. Wound Repair Regen.

[28] Davidson J (1998). Animal models for wound repair. Arch Dermatol Res.

[29] Page MJ, McKenzie JE, Bossuyt PM, Boutron I, Hoffmann TC, Mulrow CD (2021). The PRISMA 2020 statement: an updated guideline for reporting systematic reviews. BMJ.

[30] De Vries RB, Hooijmans CR, Langendam MW, van Luijk J, Leenaars M, Ritskes-Hoitinga M (2015). A protocol format for the preparation, registration and publication of systematic reviews of animal intervention studies. Evid Based Preclinical Med.

[31] Percie du Sert N, Hurst V, Ahluwalia A, Alam S, Avey MT, Baker M, et al. The ARRIVE guidelines 2.0: updated guidelines for reporting animal research. J Cerebral Blood Flow Metab. 2020;40(9):1769–77. 10.1177/0271678X20943823 PMC7430098 32663096

[32] Faggion CM, Listl S, Giannakopoulos NN (2012). The methodological quality of systematic reviews of animal studies in dentistry. Vet J.

[33] Hooijmans CR, IntHout J, Ritskes-Hoitinga M, Rovers MM (2014). Meta-analyses of animal studies: an introduction of a valuable instrument to further improve healthcare. ILAR J.

[34] Hooijmans CR, Rovers MM, De Vries RB, Leenaars M, Ritskes-Hoitinga M, Langendam MW (2014). SYRCLE’s risk of bias tool for animal studies. BMC Med Res Methodol.

[35] Guyatt GH, Oxman AD, Vist GE, Kunz R, Falck-Ytter Y, Alonso-Coello P (2008). GRADE: an emerging consensus on rating quality of evidence and strength of recommendations. BMJ.

[36] Vesterinen H, Sena E, Egan K, Hirst T, Churolov L, Currie G (2014). Meta-analysis of data from animal studies: a practical guide. J Neurosci Methods.

