# Supplementary material for: Efficacy of nano encapsulated herbal extracts in the treatment of induced wounds in animal models: a systematic review protocol
Source: Syst Rev. 2023 Nov 16;12:215. doi: 10.1186/s13643-023-02370-7 (PMC10652619; doi:10.1186/s13643-023-02370-7)
Supplement: Supplementary file 2 — Additional file 2. Pilot search strategy. [file 13643_2023_2370_MOESM2_ESM.pdf]

PILOT SEARCH STRATEGY

Findings of pilot electronic search done on 13/03/2022 in PubMed in the title or abstract or text.

Search strategy	Number of hits	Database	Potentially eligible
(((Rats[Text Word] OR mice[Text Word] OR rabbits[Text Word] OR pigs[Text Word]) AND (Nano[Title/Abstract] OR nanoparticles[Title/Abstract] OR nano-composites[Title/Abstract] OR nano-capsules[Title/Abstract] OR nano-spheres[Title/Abstract] OR nano-medicine[Title/Abstract] OR nano-liposomes[Title/Abstract] OR nanofibers[Title/Abstract] OR nanomaterials[Title/Abstract] OR nano-gels[Title/Abstract] OR dendrimers[Title/Abstract] OR cyclodextrins[Title/Abstract])) AND (Plant[Text Word] OR tree[Text Word] OR bark[Text Word] OR leaves[Text Word] OR seed Herbal crude extract[Text Word] OR plant extract[Text Word] OR herbal medicine[Text Word] OR phytochemical[Text Word] OR phytocompound[Text Word] OR secondary metabolites[Text Word] OR essential oil*[Text Word])) AND (Wound*[Title/Abstract] OR Wound healing[Title/Abstract] OR Wound model[Title/Abstract] OR excisional wound[Title/Abstract] OR incision wound[Title/Abstract] OR murine wound[Title/Abstract] OR dead space[Title/Abstract] OR burnt wound[Title/Abstract] OR infected wound[Title/Abstract] OR diabetic wound[Title/Abstract] OR splinted wound[Title/Abstract] OR cutaneous wound[Title/Abstract])	102 hits	PubMed	35
